# Prediction of Five-Year Cardiovascular Disease Risk in People with Type 2 Diabetes Mellitus: Derivation in Nanjing, China and External Validation in Scotland, UK

**DOI:** 10.5334/gh.1131

**Published:** 2022-07-28

**Authors:** Cheng Wan, Stephanie Read, Honghan Wu, Shan Lu, Xin Zhang, Sarah H. Wild, Yun Liu

**Affiliations:** 1 Department of Medical Informatic, School of Biomedical Engineering and Informatics, Nanjing Medical University, CN; 2 Women’s College Research Institute, Women’s College Hospital, Toronto, CA; 3 Institute of Health Informatics, University College London, London, UK; 4 Outpatient department, the First Affiliated Hospital, Nanjing Medical University, CN; 5 Department of Information, the First Affiliated Hospital, Nanjing Medical University, China; 6 Department of Medical Informatics, School of Biomedical Engineering and Informatics, Nanjing Medical University, CN; 7 Usher Institute, University of Edinburgh, GB; 8 Department of Information, the First Affiliated Hospital, Nanjing Medical University, No. 300 Guang Zhou Road, Nanjing, Jiangsu, 210029, China

**Keywords:** five-year cardiovascular disease risk, type 2 diabetes mellitus, derivation and external validation, routinely collected hospital data

## Abstract

**Background:**

To use routinely collected data to develop a five-year cardiovascular disease (CVD) risk prediction model for Chinese adults with type 2 diabetes with validation of its performance in a population of European ancestry.

**Methods:**

People with incident type 2 diabetes and no history of CVD at diagnosis of diabetes between 2008 and 2017 were included in derivation and validation cohorts. The derivation cohort was identified from a pseudonymized research extract of data from the First Affiliated Hospital of Nanjing Medical University (NMU). Five-year risk of CVD was estimated using basic and extended Cox proportional hazards regression models including 6 and 11 predictors respectively. The risk prediction models were internally validated and externally validated in a Scottish population–based cohort with CVD events identified from linked hospital records. Discrimination and calibration were assessed using Harrell’s C-statistic and calibration plots, respectively.

**Results:**

Mean age of the derivation and validation cohorts were 58.4 and 59.2 years, respectively, with 53.5% and 56.9% men. During a median follow-up time of 4.75 [2.67, 7.42] years, 18,827 (22.25%) of the 84,630 people in the NMU-Diabetes cohort and 8,763 (7.31%) of the Scottish cohort of 119,891 people developed CVD. The extended model had a C-statistic of 0.723 [0.721–0.724] in internal validation and 0.716 [0.713–0.719] in external validation.

**Conclusions:**

It is possible to generate a risk prediction model with moderate discriminative power in internal and external validation derived from routinely collected Chinese hospital data. The proposed risk score could be used to improve CVD prevention in people with diabetes.

## Introduction

Cardiovascular disease (CVD) is the leading cause of morbidity and mortality in China and worldwide [1,2]. People with type 2 diabetes are at approximately two- to fourfold higher risk of developing CVD than people without diabetes [3,4]. Because almost a quarter of the estimated global population of people with diabetes is in China [5], it is important to be able to support effective management of the large number of people with diabetes, especially in urban areas where diabetes prevalence is higher than in rural areas [6].

Conventionally, CVD risk prediction models are derived from cohort studies and were designed to assess the management options and develop personalized treatment strategies [6,7]. Most CVD risk prediction models [8,9,10,11,12,13] for people with type 2 diabetes were derived in cohorts from Western countries [14]. Existing CVD risk prediction models derived in Chinese populations with type 2 diabetes were derived from cohorts collected more than 20 years ago [15,16], from small data sets (less than 10,000 individuals), or from data sets that were missing important clinical characteristics at baseline [17,18]. Therefore, new CVD models for patients with type 2 diabetes in China are needed.

Application of the Observational Medical Outcomes Partnership Common Data Model (OMOP CDM) platform [19] offers a new way to create CVD risk prediction models for patients with diabetes in China using routinely collected data. Data concerning basic characteristics, diagnoses, and medications of patients were extracted from an aggregation of patient-centric health data collected in hospitals in real time can be cleaned, pseudonymized, and used for research.

Nanjing city is the capital of Jiangsu province in central-eastern China. The First Affiliated Hospital of Nanjing Medical University (NMU) is one of the best medical centers in Nanjing with a catchment area including approximately eight million people. The CDM platform of the hospital contains observational health data of more than 140,000 patients with type 2 diabetes since 2005. The NMU-diabetes database offers an opportunity to evaluate existing diabetes-specific CVD risk prediction models and to generate a new model.

These developments offer an alternative efficient approach to conducting observational research on routine care data, enabling follow-up using routine data on larger numbers of people. Risk prediction models created from such data also have the potential to be more relevant to ‘real-world’ settings.

External verification in other ethnic groups showed the differences in the diagnosis, treatment, and CVD risk factors among type 2 diabetics in different regions. It is valuable to explore whether models derived from Chinese populations might apply to other ethnic groups in other settings. Nowadays, most CVD risk prediction models were derived primarily from populations of European ancestry. It is valuable to assess whether models derived in Chinese populations might also perform well in populations of European ancestry. Scotland maintains a national population-based register, Scottish Care Information–Diabetes (SCI-Diabetes), of more than 180,000 patients with a diagnosis of type 2 diabetes that can be linked to population-based hospitalization and mortality records. It has previously been used to externally validate other CVD prediction models [20].

The aim of this study is to validate previous CVD risk prediction models for people with type 2 diabetes, then develop a five-year CVD risk prediction model for Chinese adults with type 2 diabetes using routinely collected hospital data from a large medical center in Nanjing, and validate its performance in a population of largely European ancestry identified from the population-based register of people with a diagnosis of diabetes in Scotland.

## 2 Methods

### 2.1 Study design and data source

#### 2.1.1 Derivation cohort

The derivation cohort was identified from the OMOP CDM platform in the First Affiliated Hospital of Nanjing Medical University (NMU), where we undertook a cohort study in a large number of inpatients and outpatients. Data concerning basic characteristics, diagnoses, and medications of patients were extracted from the Clinical Data Repository (CDR), an aggregation of patient-centric health data collected in hospital in real time, and went through privacy-free and cleaning treatment to map an observational medical outcomes partnership common data model (OMOP CDM, Ver. 5.0). In this research, data concerning demographic characteristics, diagnoses, and medications for more than 6.3 million patients seen as inpatients or outpatients between January 1, 2005, and December 31, 2017, were extracted. Details of the data set have been reported previously [21]. This study was approved by the Ethics Committee of the First Affiliated Hospital of Nanjing Medical University (No. 2019-SR-153), and informed consent from study participants was waived.

#### 2.1.2 Validation cohort

The external validation cohort was extracted from the Scottish Care Information (SCI) diabetes data set, which was introduced in 2000 and is populated by patient data from primary care and hospital diabetes clinics. Outcome data is obtained from linkage to the Scottish Morbidity Record (SMR01), a national hospital admission data set, and death registrations. Approval for generation and analysis of the linked data set was obtained from the Caldicott Guardians of all health boards in Scotland, the Privacy Advisory Committee of the Information Services Division of NHS National Services Scotland, and the Scottish multicenter research ethics committee.

#### 2.1.3 Participants

The derivation cohort, NMU-Diabetes, and the external validation cohort, SCI-Diabetes, both consisted of people diagnosed with type 2 diabetes between January 1, 2008 and December 31, 2017. This time frame was chosen to reflect the earliest availability of high-quality data and the most recently available data for both data sources. We defined baseline date as the date of diagnosis of type 2 diabetes using the earliest hemoglobin A1c (briefly HbA1c, ≥48 mmol/mol or 6.5%) or use of insulin or oral hypoglycemic drugs, or the first recorded diagnosis of type 2 diabetes.

We included participants who were aged between 30 and 89 years at the date of diagnosis of diabetes because there were few events in younger people and because of the complex confounding factors in older patients. Members of the cohort were followed up from baseline (date of diabetes diagnosis) until the earliest date of death, date of first CVD event (defined later), or study end date (December 31, 2017).

The cohort was restricted to people who had no previous history of CVD (as defined later) in order to enable the identification of high-risk groups that could inform approaches to primary prevention. We included individuals who were prescribed statins prior to and after type 2 diabetes diagnosis in the main analyses but conducted sensitivity analyses in subpopulations restricted to (1) people who had not been prescribed statins prior to type 2 diabetes diagnosis and (2) people who had not been prescribed statins prior to type 2 diabetes diagnosis or during follow-up. See the supplementary Figure 1 for the study criteria in the derivation cohort.

**Figure 1:**
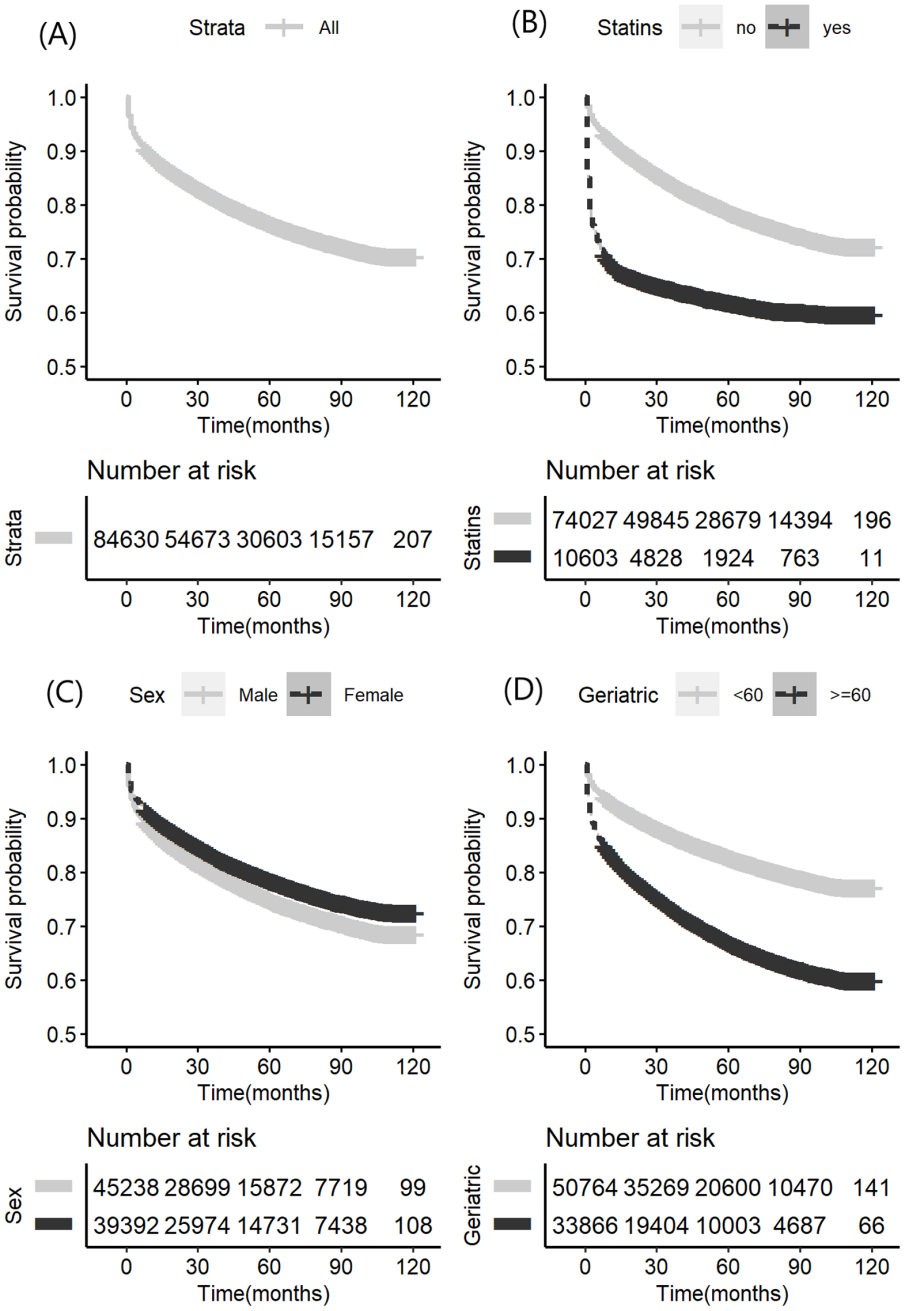
**Kaplan-Meier curve analysis in the whole derivation cohort. (A)** Survival probability for all patients in the derivation cohort. **(B)** Survival probability for all patients in the derivation cohort grouped by statins using or not. Gray line: no statins using patients. Blackline: statins using patients. **(C)** Survival probability for all patients in the derivation cohort grouped by gender. Gray line: Male. Black line: Female. **(D)** Survival probability for all patients in the derivation cohort grouped by geriatric or not. Geriatric is defined according to the clinical definition in China. Gray line: Not Geriatric, younger than 60. Black line: Geriatric, older than 60. Survival probability: the outcome modeled in the Kaplan-Meier curves are for the composite outcome defined in ‘Methods.’

#### 2.1.4 Outcome

Our outcome was cardiovascular disease, which was defined as any hospital admission or death from nonfatal myocardial infarction (International Classification of Disease [ICD-10] codes I21–I22), stroke (ICD-10 codes I60–I69), heart failure (ICD-10 code I50), cerebrovascular diseases, or transient cerebral ischemic attacks and related syndromes (ICD-10 codes G45) between baseline and December 31, 2017. CVD definitions used in derivation and external validation data sets were identical except that ICD-9 codes were used in the derivation data set for identifying the previous history of CVD due to the existence of legacy data (see Supplementary Table 1 for details).

#### 2.1.5 Predictors

The candidate predictors included in the prediction model were chosen from those used in the existing risk scores [8,9,10,11,12,13] and that were available in both NMU-Diabetes and SCI-Diabetes data sets.

In the NMU-Diabetes data set, we extracted data for demographic factors, clinical diagnoses, clinical values, and drug use. Candidate predictors of demographic factors included age (years) and sex (men/women). Candidate predictors of clinical diagnoses included rheumatoid arthritis (yes/no, ICD-10 codes M06.8, M06.9), hypertension (yes/no, ICD-10 codes I10.x), and chronic kidney disease (yes/no, ICD-10 codes N03, N11, N18). All predictor variables of clinical diagnoses were based on the latest diagnosis text in Chinese recorded in the CDM platform before entry to the cohort. Candidate predictors of clinical values included body mass index (weight/height, kg/m2), smoking status (yes/no), HbA1c (%), systolic blood pressure (mmHg), total cholesterol (mmol/L), HDL–cholesterol (mmol/L), LDL cholesterol (mmol/L), albumin-to-creatinine ratio (mg/g), albuminuria (normal, micro, macro), urine creatinine (umol/L), and estimated glomerular filtration rate (mL/min/1.73 m2). Smoking status and body mass index were extracted from medical records by natural language processing. Other clinical values were extracted from the standard clinical data in the CDM platform. Predictor values of clinical values were defined as measurements recorded closest to the date of diagnosis within 12 months before or after the baseline date of diagnosis of diabetes. Candidate predictors of drug use included antihypertensive medications (yes/no) and lipid-lowering medications (yes/no). Values of drug-use predictors were decided by the drug name in Chinese recorded in the drug exposure table in the CDM platform and the tertiary list of the China national essential drugs list (2018 edition). The descriptions of predictors were shown in Supplementary File 1 in detail.

In the SCI-Diabetes cohort, prescriptions of antihypertensive and lipid-lowering medications were defined using British National Formulary codes 2.5 and 2.12, respectively. The presence of rheumatoid arthritis was defined as patients with any prescription for disease-modifying antirheumatic drugs, defined with a British National Formulary code of 10.1.3 prior to diagnosis of diabetes. For comparability with the derivation cohort, diagnosis of hypertension was based on the presence of ICD-10 codes I10, I11, I13, and I15 in hospital records. Other predictors are defined as the same as those in the NMU-Diabetes cohort.

#### 2.1.6 Missing data

Multiple imputations were implemented using the mice algorithm in the statistical package R (Package mice version 3.7.0 mice package in R) to replace missing values in exposure and risk factor variables. Imputation models were estimated and included all the baseline covariates used in the main analysis (age, sex, smoking status, high-density lipoprotein cholesterol, low-density lipoprotein cholesterol, total cholesterol, systolic blood pressure, total cholesterol to high-density lipoprotein cholesterol ratio, HbA1c, albuminuria [normal, micro, macro], albumin-to-creatinine ratio, creatinine, eGFR), baseline medications (prescribed antihypertensive medications, prescribed statins prior to diabetes diagnosis), coexisting medical conditions (history of rheumatoid arthritis, chronic kidney, and atrial fibrillation disease), coexisting medical conditions (history of rheumatoid arthritis, chronic kidney, and atrial fibrillation disease), and survival days and the outcome event status for each endpoint. Prior (between 10 and 1 years before study entry) and post (between 0 and 1 year after study entry) averages of continuous covariates were used in the imputation. Five multiply imputed data sets were generated, and Cox models were fitted to each data set. Estimates were pooled using Marshall’s adaption of Rubin’s rules [22]. The Kolmogorov-Smirnov test was used to compare the distribution of observed versus imputed log-transformed covariates.

### 2.2 Statistical analyses and derivation and validation of the models

#### 2.2.1 Survival analysis

Grouped Kaplan-Meier analysis, a nonparametric approach, using the month as the unit of time was used to illustrate event rates. The proportional hazards (PH) assumption was checked using statistical tests and graphical diagnostics based on scaled Schoenfeld residuals. Statistical significance was defined as a p-value < 0.05, and 95% confidence intervals (CI) were calculated.

#### 2.2.2 Derivation of the models

We developed and evaluated the prediction models using existing works and performed an initial analysis based on all patients identified in the cohort. We used Cox proportional hazards regression to derive the risk prediction model. We developed a basic model that examined risk factors (age, sex, clinical diagnoses, and drug-use factors) that were easily measured in Chinese routine clinical settings and with lower data missing rates. The basic model excluded smoke status and biomarkers (eGFR, albuminuria, systolic blood pressure, LDL cholesterol, HbA1c) to test the performance of eliminating the influence of missing data on the model that used all the relevant patients on the database. Then, to maximize the power and generalizability of the results while remaining the cohort size same, we developed an extended model that used all the relevant patients on the database. We fitted full models initially, selected variables for inclusion in the model following a stepwise approach, and undertook standard model checking. The variable included in the extended model was the one associated with the smallest Akaike’s information criterion (AIC). AIC statistic estimates the relative amount of information lost by a given model and deals with the trade-off between the goodness of fit of the model and the simplicity of the model. Sex-specific models were also generated, and interactions between the predictors and sex were tested to investigate potential effect modification. AIC statistics were used to analyze the performance of the interactive model. We also considered several approaches to transforming continuous predictors, including linear, squared, log, and restricted cubic spline with four and five knots. The values of these knots can be found in Supplementary Table 2. The effect of different transformations was evaluated using Wald χ^2^. To avoid overfitting, the transformation of each continuous predictor with the highest χ^2^, if it was more than 5% higher than the χ2 value of the linear model, was chosen for inclusion in the multivariable model. We also evaluated performance in each age group (≤45, 45–60, 61–75, >76 years), persons without a previous statin prescription subgroup, the person with complete data for all predictors subgroup, and the person with complete data for some important biomarkers (albuminuria, estimated glomerular filtration rate, LDL cholesterol) subgroups. Performance was also evaluated by calculating Harrell’s C-statistics.

#### 2.2.3 Internal validation and reclassification statistics

A bootstrap approach was used in internal validation (200 replications). Bootstrap is a statistical resampling procedure that draws bootstrap samples with replacement from the original sample to introduce a random element.

We classified patients as being at high risk of CVD if their estimated five-year risk was equal to or greater than a threshold. The threshold was set as 20% according to the overall event rate of CVD in deviation cohort. Integrated discrimination improvement (IDI) was applied to compare the performance of the basic and the extended models we presented [23].

We compared predictions from our model with those from existing works, such as the American College of Cardiology/American Heart Association (ACC/AHA) Pooled Cohort Equations (PCEs) [8] for white and PECs for Africa, ADVANCE [8], Swedish NDR [12], and QRISK2 [24] for Chinese. Harrell’s C-statistic and IDI were also applied to assess how well each set of equations distinguished high-risk from low-risk patients (the threshold was set as 0.2 for cardiovascular disease).

### 2.3 External validation of the Model

The predictive performance of each derived risk score for the NMU-Diabetes cohort was assessed by internal and external validation. Discrimination of the final model was assessed using Harrell’s C-statistic during external validation. Discrimination describes the model’s ability to differentiate people who developed CVD from those who did not. Calibration was assessed in the external validation cohort using calibration slopes, calibration-in-the-large statistics, and calibration plots. Calibration slope statistics are the unitless slope of our calibration plot [25] used to evaluate the agreement between the risk prediction model and observed five-year risk using Kaplan-Meier estimates. Calibration-in-the-large statistics compare the mean predicted risk and mean observed risks.

The models were recalibrated during external validation by adjusting baseline hazard and regression coefficients of the predictors by linear regression between the predicted risks and the observed risks in the SCI-Diabetes cohort. We also evaluated performance in each age group (≤45, 45–60, 61–75, ≥76 years).

All statistical analyses were conducted in R, version 3.6.2. Details about packages and codes were shown in Supplementary file 3. The reporting of this study is in accordance with the Transparent Reporting of a multivariable prediction model for Individual Prognosis or Diagnosis (TRIPOD) guidelines.

## 3 Results

### 3.1 Derivation and validation data sets

#### 3.1.1 Characteristics of the participants

For the derivation cohort, we identified 148,624 patients with a diagnosis of diabetes from the CDM platform. We excluded 6,613 (4.45%) with a diagnosis of other diabetes, 4,297 (2.89%) with a diagnosis of type 1 diabetes, 3,622 (2.44%) aged less than 30 or missing age info at baseline, 17,244 (11.60%) who are clearly observable for drugs in the one-year period after diagnosis and have insulin prescribed in that period, 23,295 (15.67%) with a diagnosis of cardiovascular disease at baseline, and 8,923 (6.00%) with a diagnosis of type 2 diabetes before January 1, 2008. Overall, 84,630 patients were included in the derivation.

For the external validation cohort, there were 248,281 individuals diagnosed with type 2 diabetes in Scotland before December 31, 2017. Of these, 128,390 had a previous history of CVD or were diagnosed with type 2 diabetes before January 2008 and so were excluded from the analyses, leaving 119,891 (28.29%) individuals to form the external validation cohort.

Among the predictors included in the risk models, five had missing values, and the proportions of missingness were higher in NMU-Diabetes than SCI-Diabetes. In the derivation and validation cohorts, respectively, there were a total of 79,975 and 91,481 individuals with incomplete predictor data, including 2,907 and 55,638 individuals with a single incomplete predictor and a further 9,541 and 27,264 individuals with two incomplete variables (Supplementary Table 3).

#### 3.1.2 Baseline characteristics of derivation and validation cohort

Table 1 compares the characteristics of eligible patients in both cohorts. Major differences between the derivation and validation cohort included a higher prevalence of macroalbuminuria (6.8% for women and 5.1% for men) in the derivation cohort than in the validation cohort (2.9% for women and 3.6% for men), whereas the prevalence of prescribing of lipid-lowering (11.3% for woman and 13.6% for men) and antihypertensive medications (23% for woman and 24.5% for men) in the NMU-Diabetes cohort was lower than in the Scottish cohort.

**Table 1:** Characteristics of patients aged 30–89 in derivation and validation cohorts 2008–2017 [CODE: finalBaselineTable.R].


CHARACTERISTICS	DERIVATION COHORT		VALIDATION COHORT	
	
	FEMALE	MALE	FEMALE	MALE

N	39,392	45,238	51,651	68,240

Age at diagnosis, years, median (IQR)	59 (8.6)	57 (9.4)	61 (9.3)	58 (8.9)

Systolic blood pressure, mmHg, mean (SD)	131 (18.7)	130 (18.2)	137.6 (17.7)	138.1 (17.1)

Smoking status, n (%)				

no	38,840 (98.6)	28,904 (63.9)	26,061 (50.5)	28,900 (42.4)

ex	80 (0.2)	2,599 (5.7)	14,596 (28.3)	24,539 (36)

cur	472 (1.2)	13,735 (30.4)	10,994 (21.3)	14,801 (21.7)

TDL cholesterol, mmol/mol, mean (SD)	5.9 (1.6)	5.9 (1.6)	5.4 (1.2)	5.1 (1.3)

LDL cholesterol, mmol/mol, mean (SD)	3.4 (0.9)	3.4 (0.9)	3 (1.1)	2.8 (1)

HDL cholesterol, mmol/mol, mean (SD)	2.4 (0.8)	2.3 (0.8)	1.3 (0.4)	1.1 (0.3)

Glycated hemoglobin, %, mean (SD)	6.9 (1.5)	7.2 (1.7)	7.9 (2)	8.3 (2.2)

Urine creatinine (umol/L)	100.9 (66.2)	134.2 (78.6)	71.8 (18.8)	85.3 (21.8)

Albumin-to-creatinine ratio, mean (SD)	72.7 (141.5)	60.5 (122.2)	3.5 (13.5)	4.1 (14.9)

Estimated glomerular filtration rate mls/min/1.73 m2, mean (SD)	68.9 (34.9)	66 (36.5)	81.2 (19.4)	86.9 (18)

Albuminuria, n (%)				

normal	25,460 (64.6)	30,996 (68.5)	42,414 (82.1)	51,240 (75.1)

micro	11,261 (28.6)	11,919 (26.3)	7,741 (15)	14,553 (21.3)

macro	2,671 (6.8)	2,323 (5.1)	1,496 (2.9)	2,447 (3.6)

Retinopathy, n (%)				

0	37,487 (95.2)	43,416 (96)	51,643 (100)	68,225 (100)

1	1,905 (4.8)	1,822 (4)	8 (0)	15 (0)

rheumatoidarthritis, n (%)				

0	39,087 (99.2)	45,058 (99.6)	50,962 (98.7)	67,822 (99.4)

1	305 (0.8)	180 (0.4)	689 (1.3)	418 (0.6)

Atrial Fibrillation, n (%)				

0	39,210 (99.5)	44,938 (99.3)	49,806 (96.4)	65,582 (96.1)

1	182 (0.5)	300 (0.7)	1,845 (3.6)	2,658 (3.9)

Prescribed statins prior to diabetes diagnosis, n (%)				

0	34,925 (88.7)	39,102 (86.4)	36,088 (69.9)	47,204 (69.2)

1	4,467 (11.3)	6,136 (13.6)	15,563 (30.1)	21,036 (30.8)

Prescribed antihypertensive medications, n (%)				

0	30,349 (77)	34,151 (75.5)	35,987 (69.7)	48,051 (70.4)

1	9,043 (23)	11,087 (24.5)	15,664 (30.3)	20,189 (29.6)

5-year CVD event rate, n (%)	7,340 (18.63)	9,954 (22.0)	2,579 (5)	3,660 (5.4)

10-year CVD event rate, n (%)	8,048 (20.43)	10,779 (23.83)	3,647 (7.1)	5,116 (7.5)


#### 3.1.3 Incidence of cardiovascular disease

Table 1 also shows the incidence rates of cardiovascular disease by gender both in the derivation cohort and in the external validation cohort. In the derivation cohort, during median follow-up (interquartile range, IQR) of 4.75 [2.67, 7.42] years, 18,827 (22.25%) individuals developed cardiovascular disease during the study period from 10 years of observation. The 5- and 10-year CVD event rates were 22.0% and 23.83% for men and 18.63% and 20.43% for women, respectively. Kaplan-Meier survival curves for the derivation cohort are shown in Figure 1. In the survival analysis, CVD event incident risk was significantly higher among patients older than 60 (hazard ratio 1.97, 95% CI 1.92–2.03) compared with younger patients, was significantly higher among patients using statins before baseline (hazard ratio 8.2, 95% CI 7.94–8.45) compared with those who did not, and was slightly lower among female patients (hazard ratio 0.83, 95% CI 0.81–0.86) compared with male patients in the entire cohort.

#### 3.1.4 Characteristics of events

Overall, in the NMU cohort, there were 10,120 (53.75%) coronary heart disease events (I21, I22, I50), 4,043 (21.47%) cerebrovascular disease (I60–I66), and 4,664 (24.78%) other CVD events (I67, G45).

In the SCI cohort, there were 4,689 (57.87%) heart disease events, 1,718 (21.47%) cerebrovascular disease events were, and 2,356 (20.93%) other CVD events.

### 3.2 Risk prediction model development and internal assessment

#### 3.2.1 Model development

Table 2 shows the coefficients for each predictor in the basic and extended models. The basic model performed modestly overall in all patients with C-statistics of 0.716 [0.714–0.718]. The extended model identified 11 predictors associated with the risk of CVD (shown in Table 2) and had a similar C-statistic, 0.727 [0.725–0.729], to the basic model. The internal validation using a bootstrap approach showed these models were stable with C-statistics of 0.712 [0.703–0.72] for the basic model and 0.723 [0.715–0.732] for the extended model (Table 2). Results of analyses exploring improvement in model fit following the inclusion of nonlinear terms are presented in Supplementary Table 4.

**Table 2:** Adjusted hazard ratios (95% CI) for cardiovascular disease for the basic and extended models in derivation cohort.


MODEL NAME	BASIC	EXTENDED

Age at diagnosis	1.027 [1.026–1.028]***	1.025 [1.024–1.027]***

Sex:		

male	1	1

female	0.821 [0.797–0.846]***	0.86 [0.831–0.889]***

Rheumatoid Arthritis	1.63 [1.399–1.899]***	1.65 [1.416–1.922]***

Hypertension	2.873 [2.785–2.964]***	2.758 [2.672–2.847]***

Prescribed statins prior to diabetes diagnosis	2.264 [2.178–2.353]***	2.37 [2.279–2.464]***

Prescribed antihypertensive medications	0.635 [0.611–0.659]***	0.628 [0.604–0.653]***

Smoking status		

Never smoker	1	1

Ex-smoker		1.232 [1.18–1.285]***

Current smoker		1.273 [1.18–1.373]***

Albuminuria		

Normal		1

Microalbuminuria		1.096 [1.06–1.134]***

Macroalbuminuria		1.244 [1.172–1.32]***

Estimated glomerular filtration rate, mls/min/1.73 m2,		

(0, 15)		0.748 [0.631–0.888]***

（ 15, 30）		1

(30, 60)		1.146 [1.087–1.208]***

(60, 90)		1.308 [1.24–1.381]***

>90		1.354 [1.285–1.427]***

Glycated hemoglobin, %,		0.904 [0.894–0.914]***

LDL cholesterol, mmol/mol,		0.828 [0.814–0.842]***

Center	1.693	0.558

Harrell’s C-statistic	0.718 [0.716–0.72]	0.727 [0.725–0.729]

Nagelkerke’s R2	0.11	0.123

AIC	374,308.188	373,182.563

Internal Validation C-statistic (bootstrap)	0.718 [0.716–0.72]	0.727 [0.725–0.729]


*** p < 0.001.

#### 3.2.2 Sensitivity analyses

Calibration and discrimination for age-stratified subsets were shown in Supplementary Table 5. Evaluated according to the mean C-statistics, the age-stratified extended models using all factors except age performed slightly higher (with C-statistics as 0.727 [0.72, 0.734] in subgroup age lower than 45, 0.713 [0.708, 0.717] in subgroup age between 45 and 60, 0.699 [0.696, 0.703] in subgroup age between 61 and 75, and 0.673 [0.668, 0.677] in subgroup age older than 76 years, respectively) compared with the age-stratified basic models only using sex, clinical diagnoses, and drug-use factors. Totally, the age-stratified models performed better in the younger subcohort.

The sex-specific models using all factors except gender performed slightly lower (with C-statistics as 0.726 [0.723–0.729] and 0.725 [0.723–0.728] in the female and male subcohorts, respectively) compared to the results of the whole cohort. Detailed are presented in Supplementary Table 6. Interactions between the predictors and sex were consistent in the basic and the extended models (Supplementary Table 7). For each of these interactions, hazard ratios for the predictors were lower for women compared with men and raised gradually with increasing age.

In sensitivity analyses conducted in the subcohort of persons without a previous statin prescription (*N* = 74,027), model coefficients were consistent with the whole NMU-Diabetes cohort (Supplementary Table 8). In the subcohort of persons with complete data for all predictors (*N* = 2,877) (see Supplementary Table 8), hazard ratios for nonusers of statins were consistent with the whole NMU cohort. Five-year CVD incidence in the subcohort for people with complete data for all predictors was 36.32 %, nearly twice as high as the whole cohort. Hazard ratios for rheumatoid arthritis and the use of statins were in the opposite direction from those of the whole cohort. The adjusted hazard ratio for three biomarker-related predictors (albuminuria, estimated glomerular filtration rate, or LDL cholesterol), included in the extended model in derivation cohort but with high missing rate, are explored in the subcohort with no missing values.

#### 3.2.3 Internal validation and reclassification statistics

The internal validation using a bootstrap approach showed these models were stable with C-statistics of 0.712 [0.703–0.72] for the basic model and 0.723 [0.715–0.732] for the extended model (Table 2). The calibration slopes were 1.04 [1.04, 1.04] and 1.038 [1.038, 1.038], and estimates of calibration-in-large were 0.019 and 0.018 for the basic and the extended models, respectively, which means that the two models performed well in cohorts similar to the derivation cohort.

With a 20% threshold for high risk of developing CVD, the extended model classified 15.07% of the cohort as high risk, capturing 12,751 (73.73%) of the subsequent CVD events. In comparison, 14.61% of the cohort were classified as high risk by the basic model, capturing 12,362 (71.48%) of the CVD events. The IDI of the extended model compared to the basic model was 1.75% [1.64%–1.85%].

### 3.3 Validation of existing works

Reclassification statistics of comparison between the NMU extended model and other models (PEC for Africa, ADVANCE, Swedish NDR, and QRISK2 for Chinese) were shown in Supplementary Table 10.

Of the 38,743 patients classified as high risk (risk of at least 20% over five years) with the PCE score (using white people equation), 22,261 (57.5%) would be reclassified at low risk with the NMU extended model. The five-year observed risk among these reclassified patients was 13.08% [12.92%–13.24%]—that is, below the 20% threshold for high risk.

Among the 37,095 patients classified as high risk with NMU extended model, QRISK2 for Chinese model reclassified the lowest (11,747) as low risk, and ADVANCE reclassified the highest (36,204) as low risk. The five-year observed risk among those patients reclassified as low risk with QRISK for Chinese was 48.72% [46.58%–50.88%]—that is, above the 20% threshold for high risk and highest in comparison.

The annual incidence rate of cardiovascular events among those with an NMU extended model score of at least 20% was 87.06 per 1,000 person-years (95% confidence interval 86.61–87.50) for men and 62.24 per 1,000 person-years (61.90–62.57) for men. Both these figures are higher than the annual incidence rate for patients identified as high risk with PCE score (using the white people equation). The annual incidence rate for these patients was 68.43 per 1,000 person-years (68.08–68.78) for men and 56.04 (55.74–56.35) for women. In other words, at the 20% threshold, the population identified by NMU extended model was at higher risk of a CVD event than the population identified by the PCE score.

### 3.4 External validation in the SCI-Diabetes cohort

In the validation cohort, during median follow-up (IQR) of 4.75 [2.67, 7.42] years, 8,763 (7.31%) individuals in the SCI-Diabetes cohort developed cardiovascular disease CVD during the study period from 10 years of observation. The 5- and 10-year CVD event rates were 5.4% and 7.5% for men and 5.0% and 7.1% for women, respectively. Supplementary Table 11 shows the associations between variables selected in the NMU-Diabetes basic and extended models and the risk of incident CVD in the SCI-Diabetes cohort. For the basic model, hazard ratios were similar in the two cohorts, with the exception of antihypertensive medication use. For the extended model, hazard ratios for eGFR, Hemoglobin AIC, LDL, and lipid-lowering medication use were in different directions.

**Figure 2:**
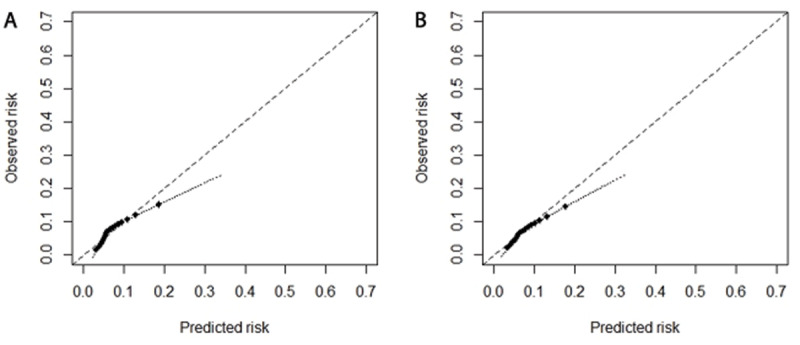
**Calibration plots for observed versus predicted five-year risk of CVD as estimated using the basic and the extended models in the derivation cohort.** Gray dashed line reflects perfect agreement between observed and predicted risk. **(A)** Calibration plots using the basic model. **(B)** Calibration plots using the extended model.

In the external validation, C-statistics were 0.691 [0.688–0.694] for the basic model and 0.714 [0.71–0.717 ] for the extended model. Measures of calibration and discrimination for subsets within the external validation cohort yielded results similar to those of the main analyses (Supplementary Table 12), and calibration plots are presented in Figure 2. For calibration, applying the basic model and the extended model to the external validation cohort by adjusting the linear predictor gave a C statistics of 0.65 [0.646, 0.654] and 0.634 [0.63, 0.637] for CVD, respectively, and good calibration with the calibration slope 1.009 [1.008, 1.01] and 1.116 [1.115, 1.117] for CVD. Overall, the basic model performed better than the extended model in the external validation cohort, but both models tended to overestimate risk. For both models, C-statistics values decreased after stratification by age, particularly in older age groups.

## 4. Discussion

We developed a five-year CVD disease risk score in people with new-onset diabetes identified from the database of a tertiary hospital in China who were found to be at high risk of developing CVD. The risk score was compared with previous CVD scores and performed moderately well in external validation in a Scottish population–based cohort.

### 4.1 Main findings

There are three main contributions of this paper. First, in this study we developed the risk models for high-risk patients using routinely collected clinical data from a large medical center. This permits better identification of patients at high risk among the large number of patients who visit large medical centers. The average incidence of CVD in these patients was higher than in population-based survey data. For example, the 10-year Kaplan-Meier ASCVD rate reported as 4.6% for men and 2.7% for women in the China-PAR Project, which is much lower than the 10-year CVD event rate (23.8% for men and 20.4% for women) in the NMU-Diabetes cohort. Although the median follow-up of the NMU-Diabetes cohort in this study, 4.75 [2.67, 7.42], is not the longest retrospective cohort in China, the median follow-up of the cohort will increase with the routinely collected process in the hospital. Therefore, it is valuable to develop a CVD risk prediction model based on longterm follow-up clinical diagnosis and treatment data extracted from top-level hospitals in China. Through this study, high-risk patients can be more effectively identified, and targeted early interventions and focused interventions can be carried out in the large medical centers.

Second, in this prospective study, we provide a new attempt to leverage clinical big data in China to help to reduce health inequalities in Western countries. In China, large medical centers have accumulated considerable clinical diagnosis and treatment data, which have the potential to offer a valuable resource for risk prediction to support individual patient management, observational research, and recruitment to clinical trials. The NMU-Diabetes cohort provided a good reference for analyzing the risk of CVD in diabetic patients in China’s eastern developed regions (according to China’s 2021 census data, which accounts for 40% of China’s total population). In contrast, in the study of the prediction of cardiovascular disease risk in people with type 2 diabetes mellitus, many existing works have studied the performances of the models established in Western countries in China, but few studies have tried to do it in reverse. Therefore, our work provides a new attempt to leverage huge clinical data in China to help to reduce health inequalities in other countries.

Third, we provide two models to better predict patients’ risk of CVD according to their visiting history. The basic model of CVD risk prediction tries to eliminate bias caused by differences in test results between different hospitals. Due to China’s current medical system, patients are free to choose hospitals, and their medical test results might have a high missing rate. Furthermore, there is also a lack of high-quality guarantees for the comparability of the results of medical tests between different hospitals because of the insufficient level of medical development. For example, in the 2020 Nanjing Medical Laboratory Intermural Quality Assessment, it was found that some hospitals’ cholesterol-related test results could not meet the relevant quality standards. Meanwhile, we provide an extended model that incorporates test results to provide more accurate risk prediction for patients who visit one hospital commonly and do medical tests in the same hospital. Compared with the existing equation for cardiovascular risk, the extended model allows more accurate quantification of risks for individual patients by incorporating important additional clinical conditions (including clinical diagnoses, clinical values, and drug use such as rheumatoid arthritis, chronic kidney disease, hypertension, antihypertensive medications, lipid-lowering medications, etc.).

### 4.2 Predictors inclusion and exclusion

According to the information available in the routinely collected data set, we retained as many predictors used by existing algorithms (QRISK2, ADVANCE, PCE, Swedish NDR, etc.) as possible.

We have produced two main final models: the basic model, which includes age, sex, clinical diagnoses, and drug-use factors that may be more suitable for patients admitted for the first time, and the extended model, which includes smoke status and biomarkers (eGFR, albuminuria, systolic blood pressure, LDL cholesterol, HbA1c) after multiple imputations for missing data that may be more suitable for patients who often visit the hospital where longitudinal repeated biomarkers values are likely to be available.

Although adopted in many risk score studies, predictors such as BMI, family history of CVD, and economic status were not included in the candidate predictors of this study. BMI is excluded as a risk factor because of the limited number of records in CDR (only 405 records) and their high values (mean ± SD 36.89 ± 8.59 kg/m2) suggesting biased recording. Few other hospitals in China record BMI limiting its value as a predictor. Although family histories of CVD were recorded in more than 50,000 patients’ admission notes in Chinese text, the structured results of family history require special complicated natural language processing and are not available in this study. Socioeconomic status is excluded as a risk factor because it was totally not recorded in the EHR system. Although some existing work determined the economic status of patients through self-report or zip code matching, it is not suggested in the EHR standard in China until now.

### 4.3 Comparisons with the literature

Hemoglobin AIC had a statistically negative correlation with incident CVD in the NMU-Diabetes cohort similar to the association reported for women in the five-year CVD risk prediction study in Hong Kong. For example, in Model 1 proposed by the Hong Kong Cohort Study [26], the hazard ratio for Hemoglobin AIC was 0.73 [0.63, 0.85] in women and 1.25 [1.09, 1.42] in men. In contrast, Hemoglobin AIC is positively associated with CVD risk in people with type 2 diabetes in Scotland [20], the United States [27], and the United Kingdom [28,29]. Further research is required to investigate potential explanations for this discrepancy, which may be related to the duration of diabetes that can be difficult to establish in the absence of regular screening.

In terms of the effects of drug use on CVD risk, the effects of lipid-lowering drugs are consistent with existing studies [26,30]. Statin prescription at diagnosis of diabetes (12.53%) appears to be low in NMU cohort compared to that (30.53%) in the Scottish cohort. Mean LDL in the whole NMU cohort and the subcohort with a diagnosis of hypertension are 3.39 and 3.39 mmol/L, respectively, considerably higher than 2.84 and 2.66 in comparable Scottish groups. In contrast, prescribed statins prior to diabetes diagnosis became a protective factor (OR 0.934 [0.798–1.093] in the basic model and 0.969 [0.827–1.135] in the extended model) among the subcohort of patients with complete data. Patients with complete data visited the medical center more than other patients, and the incidence rate (36.32%) of the subcohort is much higher than the whole cohort (18.63% for female and 22% for male). According to ‘2017 China Diabetes Prevention and Control Guidelines [6],’ high risk is adults with one or more of the 12 risk factors, such as older than 40 years, hypertension or undergoing antihypertensive treatment, and dyslipidemia or receiving lipid-lowering therapy. Target LDL-C for people with diabetes is less than 2.6 mmol/L, which is much lower than the values observed result in the NMU cohort. The proposed risk score in this paper could potentially be used to target prescribing of statins to people at particularly high risk of CVD.

In some existing research, hypertension-related predictors are defined in different ways. For example, treated hypertension (diagnosis of hypertension and treatment with at least one antihypertensive drug) was combined as one predictor in QRISK3 and resulted in adjusted ORs of 1.66 [1.60–1.73]. Systolic blood pressure of ≥150 mm Hg and diastolic blood pressure of ≥90 mm Hg were used as two predictors in research using cohorts from the First Affiliated Hospital of Zhengzhou University, Henan, China [17]. In the NMU-Diabetes cohort, 7,225 patients with hypertension diagnosis were not taking antihypertensive drugs in one year. Accordingly, the diagnosis of hypertension and antihypertensive drug prescription was used as two separate predictors on the basis of advice from clinical experts. In both models, the diagnosis of hypertension was a risk factor, and antihypertensive medicine prescription was a protective factor. This suggests that in patients with diabetes mellitus complicated with hypertension, antihypertensive drug therapy has a significant protective effect on reducing the risk of CVD.

Chronic kidney disease–related risk factors like albumin-to-creatinine ratio, albuminuria (normal, micro, macro), urine creatinine, and estimated glomerular filtration rate (eGFR) act as positive or negative risk factors in different subcohorts. Among the patients in three subcohorts in the derivation cohort with no missing albuminuria, with no missing estimated glomerular filtration rate, or with no missing LDL cholesterol, incidence rates of five-year CVD are, respectively, 29.5%, 30.5%, and 41.4% higher than the incidence of the whole NMU-Diabetes cohort. All of them showed positive risk OR adjusted by age and gender. Among the subcohort for patients without prescribed statins used prior to diabetes diagnosis and the subcohort for the person with complete data for all predictors, LDL cholesterol showed a negative risk effect, and estimated glomerular filtration rate remained a positive risk predictor. The adjusted hazard ratio of chronic kidney disease stage (defined by eGFR value range) was presented in gender-specified extended mode, in line with other published studies. So the chronic kidney disease stage (defined by the eGFR value range) is suggested to be used in risk prediction instead of the values of LDL cholesterol.

#### 4.3.1 Overall performance

Differences in follow-up time, cohort size, and CVD definition make it difficult to make direct comparisons with previous similar studies, and the performance of our model was similar to previous models. For example, the C-statistic for internal validation of the China-PAR Project (Prediction for ASCVD Risk in China) 10-year ASCVD risk prediction model was 0.794 [0.775–0.814] [31]. In a study from Hong Kong [30], a five-year ASCVD risk prediction model had a C-statistic of 0.705 [0.693, 0.716] in internal validation. The definition of CVD in the Hong Kong cohort was similar to ours, but the sudden death for unknown reason was also included in the outcome. Previous validation of risk scores developed in populations of European ancestry among Chinese populations have only shown moderate performance, even after recalibration leading to recommendations that ethnic-specific risk scores are developed [32,33,34].

When we evaluate the performance of some existing models using similar outcome and similar predictors available in the NMU cohort, the ACC/AHA Pooled Cohort Equations (PCE for white and PCE for Africa), ADVANCE, Swedish NDR, and QRISK2 all did not perform very well in the deviration cohort. All of them underestimate the CVD risk of patients in the NMU cohort.

### 4.4 Differences in risk of cardiovascular disease demonstrated in external validation

As expected, we needed to recalibrate our CVD risk model as part of the external validation. The difference in the rate of CVD between the two cohorts is more than 14%. It might be caused by the difference in the cohort data sources. The NMU-Diabetes cohort collected the data from a tertiary hospital in Jiangsu province. The patients in the hospital are more serious than those in other hospitals in Nanjing. Patients of this cohort showed moderate adherence, and some patients cannot maintain regular visits to the hospital. The SCI-Diabetes cohort is populated by patient data from primary care and hospital diabetes clinics. The incidence rate of CVD in the SCI-Diabetes cohort decreased 7.3% because of their years of effort to control CVD risk in diabetes. Furthermore, we noted that the component of the composite outcome differed in the NMU and Scottish cohorts. In the NMU-Diabetes cohort, the proportion of the outcome made up by coronary disease not further specified is 29.92%, which is much higher than in the Scottish cohort (10.2%).

### 4.5 Limitations

Key limitations of the NMU-Diabetes cohort were limited data on BMI and socioeconomic status, which meant that these could not be included in the risk models. The proportions of missing data for biochemical predictors, such as systolic blood pressure and LDL cholesterol, were higher in the NMU-Diabetes cohort than in the Scottish cohort. But both the basic model and extended model derived from the cohort with data after imputation performs better than those from the cohort without missing data. Risk prediction based on patient medication records and related blood test results still yielded adequate performance of the risk score. The data were obtained from a single hospital and therefore may not be representative of the wider population. We will try the validation of different hospitals in China in future work.

### 4.6 Clinical implementation

The results of this study can be easily embedded into existing electronic clinical systems to support clinician decision-making. It also provides an example of how to use the routinely collected clinical data to create useful prediction models for chronic disease complications. There are some patients in whom the proposed models should not be calculated, including patients who were diagnosed as type 2 diabetes and type 1 diabetes at different times, and those with preexisting cardiovascular disease.

The study results further suggest the importance of multicenter clinical electronic medical record integration, and we recommended that patients’ smoking status and BMI be systematically collected and recorded in a more useful format than free text in the EHR in China. Further research is needed to establish whether the wider use of risk scores improves the quality of care and outcomes for people with diabetes. The external validation will provide a reference for the treatment of patients with type 2 diabetics, and maybe with rare subtypes of diabetes if the risk models based on a big population in China perform well in other ethnic groups.

In conclusion, we developed a five-year CVD risk model in Chinese people diagnosed with type 2 diabetes based on a large, retrospective cohort study in a population treated in tertiary care. The risk score performed moderately well in both internal and external validation. We conclude that although there is scope to further improve risk scores for incident CVD among people with type 2 diabetes, Chinese databases have the potential to provide a valuable source of data for the development of future risk scores for populations from diverse ethnic groups.

## Additional Files

The additional files for this article can be found as follows:

10.5334/gh.1131.s1 Supplementary File 1. Supplementary Files 1 to 3.

10.5334/gh.1131.s2 Supplementary File 2. Supplementary Figure 1 and Tables 1 to 12.

## Author Information

The NMU data underlying this article will be shared at reasonable request to the corresponding author Y. L. The SCI data underlying this article will be shared at reasonable request to the corresponding author S. W.
