# Supplementary material for: Prediction of Five-Year Cardiovascular Disease Risk in People with Type 2 Diabetes Mellitus: Derivation in Nanjing, China and External Validation in Scotland, UK
Source: Glob Heart. 2022 Jul 28;17(1):46. doi: 10.5334/gh.1131 (PMC9336685; doi:10.5334/gh.1131)
Supplement: Supplementary File 1. — Supplementary Files 1 to 3. [file gh-17-1-1131-s1.pdf]

Supplementary Methods and Results

Prediction of ten-year cardiovascular disease risk in people with type 2 diabetes mellitus: derivation in Nanjing, China and external validation in Scotland, UK

Cheng Wan*, Steph Read*, P, Honghan Wu, Shan Lu, Xin Zhang, Sarah H Wild, Yun Liu

Supplementary File 1. The description of measurement of predictors

Supplementary File 2. Multiple imputation procedure for the missing data

Supplementary File 3. R packages used in the study

Supplementary Table 1. Definition of CVD by ICD-9 and ICD-10 used in derivation cohort and validation cohort

Supplementary Table 2. The knots calculated by restricted cubic spline for continuous predictors transformations in the derivation cohort

Supplementary Table 3. Extent of missing data within each incomplete variable

Supplementary Table 4. Impact of inclusion of non-linear terms for continuous predictors in a univariate Cox regression models (Wald $\chi 2$) in derivation cohort

Supplementary Table 5· Calibration and discrimination of internal validation in the whole NMU-cohort

Supplementary Table 6. Adjusted hazard ratios (95% CI) for cardiovascular disease for sex-specific models in derivation cohort

Supplementary Table 7· Adjusted hazard ratio for interactions between the predictors and sex in derivation cohort

Supplementary Table 8· Adjusted hazard ratio for two sub cohorts in derivation cohort

(1) the subcohort for person without prescribed statins using prior to diabetes diagnosis and

(2) the subcohort for person with complete data for all predictors

Supplementary Table 9 Adjusted hazard ratio for three sub cohorts in derivation cohort with no missing Albuminuria, Estimated glomerular filtration rate, or LDL cholesterol.

(1) the subcohort for person with complete data for Albuminuria

(2) the subcohort for person with complete data for Estimated glomerular filtration rate

(3) the subcohort for person with complete data for LDL cholesterol

Supplementary Table 10 • Number of individuals and events in people with 10-year predicted risks < cutoff or ⩾cutoff as estimated using each risk score in the population of people with type 2 diabetes in derivation cohort

cutoff = 10% or 20%

Supplementary Table 11·Adjusted hazard ratio and performance in external validation in the SCI-diabetes cohort

Supplementary Table 12· Calibration and discrimination of external validation

Supplementary Fig 1. Study criteria in derivation cohort

Supplementary File 1. The description of candidate predictors included in the deviration and validation cohorts

Age (years): onset age of diabetes in both cohorts.

Sex (men/women): gender of patients recorded at baseline in both cohorts.

Smoking status: extracted from the text of admission record and outpatient records in NMU-diabetes cohort and the values of "obacco consumption at date of contact including smoking, chewing and use of other nicotine substances lifestyle-smoker" in SCI-diabetes cohort.

Hemoglobin A1C (%): in both cohorts, all HbA1c values were converted to the DCCT standard values using the formula: HbA1c (DCCT) = 0.923 HbA1c (Mono-S) + 1.345;

Systolic blood pressure(mmHg), LDL cholesterol(mmol/L), total cholesterol(mmol/L), HDL–cholesterol, albumin-to-creatinine ratio(mg/g), and urine creatinine (umol/L): in both cohorts, the values extracted from CDM were used.

Estimated glomerular filtration rate (mL/min/1.73 m2, eGFR), in both cohorts, eGFR is calculated using the formula:

Female 141*min(creatinine/0.7,1)^ (-0.329)*max(creatinine/0.7,1)^(-1.209) *0.993^age*1.018

Male: eGFR=141*min(creatinine/0.9,1)^ (-0.411)*max(creatinine/0.9,1)^(-1.209) *0.993^age

total-to-HDL–cholesterol ratio (TC:HDL): in both cohorts, TC:HDL is calculated using the formula: TC:HDL=total cholesterol / HDL–cholesterol.

Albuminuria(normal, micro, macro), the albuminuric clinical status at baseline, is extracted directly from the SCI-diabetes database and is calculated by the value of ACR according to formular in NMU-diabetes cohort: albuminuria = ifelse(ACR>300, "macro", ifelse(ACR>=30, "micro", "normal"))

Use of medications, such as antihypertensive medications (yes/no) and lipid-lowering medications(yes/no), was defined by the history of prescriptions.

History of diseases, such as rheumatoid arthritis (yes/no, ICD-10 codes: M06.8, M06.9), hypertension (yes/no, ICD-10 codes: I10.x), and chronic kidney disease (yes/no, ICD-10 codes: N03, N11,N18) was defined by the history of diagnosis, regardless of the level of severity.

Supplementary File 2. Multiple imputation procedure for the missing data

Multiple imputation was implemented using the mice algorithm in the statistical package R, to replace missing values in exposure and risk factor variables. Imputation models were estimated included:

1. All the baseline covariates used in the main analysis (age, sex, smoking status, high density lipoprotein cholesterol, low density lipoprotein cholesterol, total cholesterol, systolic blood pressure, total cholesterol to high density lipoprotein cholesterol ratio, hemoglobin A1C, albuminuria(normal, micro, macro), albumin-to-creatinine ratio, creatinine, eGFR, diagnosis of);

2. Prior (between 10 and 1 years before study entry) and post (between 0 and 1 year after study entry) averages of continuous covariates in the main analysis;

3. Baseline medications (Prescribed antihypertensive medications, Prescribed statins prior to diabetes diagnosis);

5. Coexisting medical conditions (history of rheumatoid arthritis, chronic kidney and atrial fibrillation disease);

6. The survival days and the outcome event status for each endpoint analysed in the data.

Non-normally distributed variables were log-transformed for imputation and exponentiated back to their original scale for analysis. Five multiply imputed datasets were generated, and Cox models were fitted to each dataset. Coefficients were combined using Rubin’s rules. The Kolmogorov-Smirnov test was used to compare the distribution of observed versus imputed log-transformed covariates.

Supplementary File 3. R packages used in the study

R packages used in the study include the following:
"mice", "RMySQL","tidyverse", "plyr", "foreign","stringr","lubridate","epicalc", "survival","survminer","rms","ggplot2","pec"
Detail codes used in this article will be shared at reasonable request to the first author Cheng Wan.
