# Supplementary material for: Prediction of Five-Year Cardiovascular Disease Risk in People with Type 2 Diabetes Mellitus: Derivation in Nanjing, China and External Validation in Scotland, UK
Source: Glob Heart. 2022 Jul 28;17(1):46. doi: 10.5334/gh.1131 (PMC9336685; doi:10.5334/gh.1131)
Supplement: Supplementary File 2. — Supplementary Figure 1 and Tables 1 to 12. [file gh-17-1-1131-s2.pdf]

Supplementary Table 1. Definition of CVD by ICD-9 and ICD-10 used in derivation cohort and validation cohort

TYPE of ICD	ICD CODES
CVD ICD-10	"I21","I22","I50",I60,"I61","I62","I63","I64","I65","I66", "I67","I68","I69","G45"
CVD ICD-9	"410","411","412","413","414",”428”,"430","431","432","434","435","436","437","438"

Supplementary Table 2. The knots calculated by restricted cubic spline for continuous predictors transformations in the derivation cohort

Candidate Predictors	Knots of rcs3	Knots of rcs4
**ACR**	4.3,10.2,31,350.2	4.3,8.4,16.3,51,350.2
**Glycated hemoglobin**	5.4,6.3,7.4,11	5.4,6.1,6.8,7.8,11
**Systolic blood pressure**	104,122,135,162	104,120,130,140,162
**HDL cholesterol**	0.73,1.05,1.29,1.82	0.73,0.99,1.17,1.37,1.82
**LDL cholesterol**	1.66,2.65,3.34,4.59	1.66,2.48,2.99,3.54,4.59
**TDL cholesterol**	2.94,4.27,5.18,6.86	2.94,4.03,4.72,5.44,6.86
**Creatinine**	35.72,81.60,137.20,271.70	35.72,70.30,106.60,156.40,271.70
**Age**	37,52,62,78	37,49,57,65,78
**TDL to HDL ratio**	2.51,3.56,4.45,6.42	2.51,3.36,3.98,4.73,6.42

Supplementary Table 3. Extent of missing data within each incomplete variable

Characteristic	NMU-diabetes<br>No· incomplete (%)	SCI-diabetes<br>No· incomplete (%)
Smoking status	57401(67.8)	8033(6.7)
Glycated haemoglobin	60016(70.9)	7194(6)
Systolic blood pressure	58046(68.6)	5515(4.6)
LDL-cholesterol	79937(94.5)	75052(62.6)
Estimated Glomerular Filtration Rate	72495(85.7)	7314(6.1)
Albuminuria	75862(89.6)	61744(51.5)

Supplementary Table 4. Impact of inclusion of non-linear terms of for continuous predictors in a univariate Cox regression models (Wald χ2) in derivation cohort

Predictor	Linear	Log	Squared	Rcs4	Rcs3
Hemoglobin AIC	1005.9	1175.3	1304.44	1388.73	1406.66
Systolic blood pressure	247.66	244.78	246.8	256.85	246.24
HDL cholesterol	67.08	72.75	73.16	67.96	67.38
LDL cholesterol	209.81	232.11	232.48	252.39	253.68
TDL to HDL ratio	348.05	407	480.15	415.52	415.58
TDL cholesterol	149.67	169.3	160.85	190.43	189.61
Creatinine	198.89	219.56	220.29	225.89	225.55
age	2841.46	2495.9	3083.93	3083.33	3085.35

Linear means no transforming continuous predictors. And rest four transformations are defined as the following: Log means log(x), Squared means squared(x^2), Rcs4 means restricted cubic spline with four knots, and Rcs5 means restricted cubic spline with five knots.

Supplementary Table 5· Calibration and discrimination of internal validation in the whole NMU-cohort

	Model Name	Age-group(years)	Observed 5-year risk	Predicted 5-year risk, %, median (IQR)	Calibration in the large	Calibration slope	C statistic (discrimination)
1	Basic	overall	0.185(0.134,0.303)	0.203(0.148,0.328)	0.019	1.04(1.04,1.04)	0.716(0.713,0.718)
2	Extended	overall	0.185(0.125,0.305)	0.202(0.137,0.329)	0.018	1.038(1.038,1.038)	0.727(0.725,0.729)
3	Basic	<=45	0.093(0.082,0.11)	0.1(0.091,0.116)	0.004	0.961(0.959,0.963)	0.706(0.699,0.713)
4	Extended	<=45	0.094(0.069,0.15)	0.103(0.079,0.154)	0.007	1.011(1.009,1.012)	0.727(0.72,0.734)
5	Basic	46–60	0.131(0.11,0.226)	0.145(0.127,0.227)	0.011	0.964(0.962,0.965)	0.7(0.696,0.704)
6	Extended	46–60	0.139(0.105,0.218)	0.147(0.112,0.23)	0.01	1.027(1.027,1.027)	0.713(0.708,0.717)
7	Basic	61-75	0.219(0.161,0.399)	0.258(0.204,0.404)	0.035	0.978(0.976,0.979)	0.684(0.68,0.688)
8	Extended	61-75	0.221(0.147,0.39)	0.256(0.19,0.392)	0.032	0.989(0.987,0.99)	0.699(0.696,0.703)
9	Basic	76-89	0.344(0.296,0.525)	0.432(0.376,0.63)	0.088	1.069(1.069,1.07)	0.657(0.652,0.662)
10	Extended	76-89	0.357(0.272,0.504)	0.443(0.343,0.604)	0.082	1.069(1.069,1.07)	0.673(0.668,0.677)

Supplementary Table 6. Adjusted hazard ratios (95% CI) for cardiovascular disease for sex-specific models in derivation cohort

Sub Cohort Name	NMU_female	NMU_male	NMU_female	NMU_male
Model Name	basic_Female	basic_Male	extended_Female	extended_Male
N	39392	45238	39392	45238
N event	7340	9954	7340	9954
Incidence rate	0.1863	0.22	0.1863	0.22
Age at diagnosis	1.027[1.025-1.029]***	1.027[1.026-1.029]***	1.026[1.024-1.028]***	1.025[1.024-1.027]***
Hypertensive	3.125[2.978-3.279]***	2.811[2.698-2.928]***	2.935[2.795-3.082]***	2.642[2.534-2.754]***
Rheumatoid arthritis	1.703[1.396-2.077]***	1.567[1.234-1.989]***	1.702[1.395-2.077]***	1.565[1.232-1.988]***
Prescribed antihypertensive medications in one year	0.607[0.572-0.644]***	0.659[0.627-0.693]***	0.594[0.559-0.631]***	0.651[0.619-0.685]***
Prescribed Statins in one year	2.317[2.18-2.461]***	2.28[2.168-2.398]***	2.416[2.274-2.568]***	2.334[2.219-2.456]***
Smoking status				
Ex-smoker			1.085[0.885-1.33]	1.233[1.18-1.289]***
Current smoker			1.442[0.939-2.214].	1.266[1.172-1.368]***
No smoker			1	1
Albuminuria				
Microalbuminuria			1.236[1.133-1.347]***	1.259[1.159-1.367]***
Macroalbuminuria			1.131[1.075-1.191]***	1.072[1.024-1.121]**
Normal			1	1
Estimated glomerular filtration rate				
[0,15]			0.614[0.465-0.809]***	0.876[0.704-1.089]

(15, 30]			1	1
(30,60]			1.19[1.092-1.296]***	1.118[1.045-1.196]**
(60,90]			1.306[1.198-1.424]***	1.314[1.226-1.408]***
>90			1.413[1.301-1.535]***	1.313[1.227-1.404]***
Glycated hemoglobin			0.888[0.872-0.905]***	0.912[0.899-0.925]***
LDL cholesterol			0.826[0.804-0.848]***	0.829[0.81-0.848]***
AIC	147725.823	203192.709	147148.235	202457.346
center	1.83	1.815	0.523	0.688
Harrell’s C statistic	0.714[0.711-0.717]	0.713[0.711-0.716]	0.726[0.723-0.729]	0.725[0.723-0.728]

Supplementary Table 7· Adjusted hazard ratio for interactions between the predictors and sex in derivation cohort[Update according to the new result]

*** means p<0·001,** means p<0·01,* means p<0·05

Interactive Model Name	Age+Sex*Smoking status	Interactive Model Name	Age+Sex*Rheumatoid Arthritis
Age	1.032[1.031-1.033]***	Age	1.032[1.031-1.033]***
Female	0.852[0.824-0.882]***	Female	0.779[0.756-0.803]***
Ex-smoker	1.373[1.271-1.482]***	Rheumatoid Arthritis	2.065[1.627-2.622]***
Current smoker	1.262[1.209-1.318]***	Female:Rheumatoid Arthritis	0.967[0.709-1.319]
Female:Ex-smoker	0.97[0.627-1.498]	Harrell's C statistic	0.627[0.624-0.629]
Female:Current smoker	0.896[0.728-1.103]	AIC	380930.7
Harrell's C statistic	0.629[0.627-0.631]		
AIC	380851.5		
Interactive Model Name	Age+Sex*Albuminuria	Interactive Model Name	Age+Sex*Hypertensive
Age	1.031[1.03-1.033]***	Age	1.027[1.026-1.028]***
Female	0.767[0.738-0.796]***	Female	0.783[0.751-0.816]***
Microalbuminuria	1.064[1.018-1.113]**	Hypertensive	2.909[2.796-3.026]***
Macroalbuminuria	1.256[1.16-1.36]***	Female:Hypertensive	1.088[1.024-1.156]**
Female:Microalbuminuria	1.054[0.985-1.127]	Harrell's C statistic	0.692[0.69-0.694]
Female:Macroalbuminuria	0.98[0.874-1.1]	AIC	376230.5

Harrell’s C statistic	0.626[0.624-0.629]		
AIC	380932.4		
Interactive Model Name	Age+Sex*eGFR	Interactive Model Name	Age+Sex*Prescribed statins prior to diabetes diagnosis
Age	1.032[1.031-1.033]***	Age	1.03[1.029-1.031]***
Female	0.722[0.676-0.772]***	Female	0.791[0.764-0.818]***
eGFR	1.003[1.003-1.004]***	Prescribed statins prior to diabetes diagnosis	2.39[2.281-2.505]***
Female:eGFR	1.001[1-1.002]**	Female:Prescribed statins prior to diabetes diagnosis	1.037[0.964-1.115]
Harrell’s C statistic	0.629[0.627-0.631]	Harrell’s C statistic	0.663[0.66-0.665]
AIC	380704.782	AIC	379048.159
Interactive Model Name	Age+Sex*HbA1c	Interactive Model Name	Age+Sex*Prescribed antihypertensive medications
Age	1.031[1.03-1.033]***	Age	1.032[1.031-1.033]***
Female	0.963[0.822-1.129]	Female	0.788[0.761-0.816]***
HbA1c	0.871[0.859-0.884]***	Prescribed antihypertensive medications	1.022[0.976-1.071]
Female:HbA1c	0.965[0.943-0.988]**	Female:Prescribed antihypertensive medications	0.966[0.899-1.037]
Harrell’s C statistic	0.637[0.634-0.639]	Harrell’s C statistic	0.626[0.623-0.628]

AIC	380192.3	AIC	380995.8
Interactive Model Name	Age+Sex*LDL		
Age	1.031[1.03-1.032]***		
Female	0.782[0.697-0.877]***		
LDL	0.825[0.806-0.843]***		
Female:LDL	1.002[0.968-1.037]		
Harrell’s C statistic	0.633[0.63-0.635]		
AIC	380492.2		

Supplementary Table 8· Adjusted hazard ratio for two sub cohorts in derivation cohort

(1) the subcohort for person without prescribed statins using prior to diabetes diagnosis and

(2) the subcohort for person with complete data for all predictors

Sub cohort Name	No prescribed statins prior to diabetes diagnosis		Person with complete data for all predictors	
N	74027		2877	
N event	13469		1045	
Incidence rate	0.1819		0.3632	
Model name	Basic	Extended	Basic	Extended
Age at diagnosis	1.028[1.027-1.029]***	1.02[1.017-1.022]***	1.041[1.036-1.046]***	1.039[1.034-1.044]***
Sex: female	0.826[0.798-0.854]***	0.871[0.808-0.939]***	0.899[0.791-1.023]	0.913[0.792-1.053]
Hypertensive	4.161[4.019-4.307]***	3.882[3.747-4.021]***	1.579[1.391-1.793]***	1.529[1.344-1.74]***
Rheumatoid arthritis	1.856[1.581-2.178]***	1.866[1.589-2.191]***	0.615[0.197-1.917]	0.582[0.186-1.816]
Prescribed antihypertensive medications	0.409[0.388-0.432]***	0.4[0.379-0.423]***	0.592[0.504-0.697]***	0.606[0.515-0.714]***
Prescribed statins prior to diabetes diagnosis			0.934[0.798-1.093]	0.969[0.827-1.135]
Smoking status				
Ex-smoker		1.257[1.197-1.32]***		1.064[0.906-1.25]

**Current smoker**		1.245[1.136-1.365]***		0.93[0.689-1.257]
**Albuminuria**				
**Microalbuminuria**		1.313[1.222-1.411]***		1.058[0.866-1.294]
**Macroalbuminuria**		1.111[1.069-1.154]***		1.014[0.886-1.16]
**Estimated glomerular filtration rate**		1.003[1.003-1.004]***		1.001[0.999-1.003]
**Glycated hemoglobin**		0.898[0.886-0.91]***		0.919[0.884-0.955]***
**LDL cholesterol**		0.824[0.809-0.841]***		0.896[0.837-0.958]**
**AIC**	286193.436	314016.879	16385.867	16376.079
**Center**	1.658	0.783	2.289	1.546
**Harrell's C statistic**	0.712[0.709-0.714]	0.724[0.721-0.726]	0.673[0.664-0.682]	0.68[0.672-0.689]

*** means p<0·05

Supplementary Table 9 Adjusted hazard ratio for three sub cohorts in derivation cohort with no missing Albuminuria, Estimated glomerular filtration rate, or LDL cholesterol.

(1) the subcohort for person with complete data for Albuminuria

(2) the subcohort for person with complete data for Estimated glomerular filtration rate

(3) the subcohort for person with complete data for LDL cholesterol

Sub cohort Name	NMU subcohort (1)	NMU subcohort (2)	NMU subcohort (3)
**N**	8768	12135	4689
**N event**	2583	3704	1942
**Incidence rate**	0.2946	0.3052	0.4142
**Age at diagnosis**	1.042[1.038-1.045]***1.038[1.035-1.042]***	1.039[1.036-1.041]***	1.031[1.027-1.034]***
**female**	0.882[0.815-0.954]**0.87[0.796-0.949]**	0.876[0.82-0.936]***	0.978[0.893-1.071]
**Albuminuria**			
**Microalbuminuria**	1.06[0.973-1.155]	--	--
**Macroalbuminuria**	1.188[1.018-1.385]*	--	--
**Estimated glomerular filtration rate**	--	1.002[1.001-1.003]***	--
**LDL cholesterol**	--	--	1.162[1.029-1.312]*

**Harrell's C statistic**	0.648[0.643-0.654]	0.642[0.638-0.647]	0.622[0.616-0.629]

Supplementary Table 10 • Number of individuals and events in people with 10-year predicted risks < cutoff or ⩾cutoff as estimated using each risk score in population of people with type 2 diabetes in derivation cohort

cutoff = 20%

	PCE_WHITLE	PCE_AFRICA	ADVANCE	Swedish NDR	QRISK2 _Chinese
**patients classified as high risk with the score for comparing, N**	38743	26442	1318	1296	38639
**reclassified at low risk with NMU extended model, N(%)**	22261 (57.5%)	14423 (54.5%)	427 (32.4%)	179 (13.8%)	29572 (76.5%)
**5 year observed risk among these reclassified patients**	13.08% [12.92% - 13.24%]	13.23% [13.07% - 13.39%]	14.77% [14.62% - 14.92%]	15.73% [15.61% - 15.85%]	16.02% [15.61% - 16.43%]
**patients classified as high risk with NUM extended model, N**	37095	37095	37095	37095	37095
**reclassified as low risk with the score for comparing**	20613 (55.6%)	25076 (67.6%)	36204 (97.6%)	35978 (97%)	11747 (56.4%)
**5 year observed risk among these reclassified patients**	38.44% [35.79% - 41.09%]	38.05% [35.49% - 40.61%]	38.08% [35.5% -40.66%]	37.86% [35.35% - 40.37%]	48.73% [46.58% - 50.88%]

Supplementary Table 11 Adjusted hazard ratio and performance in external validation in the SCI-diabetes cohort

Model Name	Basic	Extended
N	119891	
N event	6239	
Incidence rate	5.2%	
Age at diagnosis	1.059[1.056-1.061]***	1.058[1.055-1.061]***
Sex:		
male	1	1
female	0.757[0.719-0.797]***	0.767[0.728-0.809]***
Smoking status		
no		1
ex		1.998[1.874-2.131]***
cur		1.192[1.124-1.265]***
Albuminuria		
normal		1
micro		1.321[1.245-1.402]***
macro		1.562[1.395-1.748]***
Estimated glomerular filtration rate		0.992[0.991-0.994]***

**Glycated hemoglobin**		1.03[1.018-1.043]***
**LDL cholesterol**		1.029[1.002-1.056]*
**Rheumatoid Arthritis**	1.542[1.253-1.897]***	1.523[1.238-1.874]***
**Hypertensive**	1.409[1.32-1.505]***	1.406[1.317-1.502]***
**Prescribed statins prior to diabetes diagnosis**	0.991[0.939-1.047]	0.969[0.915-1.026]
**Prescribed antihypertensive medications**	1.024[0.969-1.082]	1.045[0.988-1.105]
**Center**	3.304	3.141
**Harrell's C statistic**	0.691[0.688-0.694]	0.714[0.71-0.717]

*** means p<0·05

Supplementary Table 1· Calibration and discrimination of external validation

Model Name	Age -group (years)	Observed 5-year risk	Predicted 5-year risk, %, median (IQR)	Calibration in the large	Calibration slope	C statistic (discrimination)
Basic	overall	0.07(0.037,0.089)	0.058(0.045,0.083)	0.003	1.009(1.008,1.01)	0.65(0.646,0.654)
	30–45	0.018(0.017,0.02)	0.011(0.01,0.013)	-0.008	0.62(0.62,0.62)	0.589(0.57,0.609)
	46–60	0.038(0.035,0.042)	0.035(0.032,0.039)	-0.003	0.925(0.925,0.925)	0.572(0.564,0.58)
	61-75	0.077(0.068,0.084)	0.08(0.074,0.092)	0.009	0.964(0.962,0.966)	0.547(0.541,0.553)
	76-89	0.175(0.163,0.193)	0.22(0.207,0.243)	0.047	1.213(1.213,1.213)	0.538(0.532,0.545)
Extended	overall	0.066(0.042,0.086)	0.064(0.05,0.091)	0.008	1.116(1.115,1.117)	0.634(0.63,0.637)
	30–45	0.019(0.017,0.021)	0.015(0.014,0.017)	-0.004	0.793(0.793,0.793)	0.555(0.536,0.574)
	46–60	0.039(0.035,0.044)	0.038(0.034,0.043)	-0.001	0.98(0.98,0.98)	0.56(0.553,0.568)
	61-75	0.073(0.067,0.082)	0.083(0.076,0.093)	0.01	1.128(1.128,1.128)	0.539(0.533,0.544)
	76-89	0.178(0.166,0.192)	0.22(0.206,0.236)	0.042	1.196(1.196,1.196)	0.532(0.525,0.538)

Supplementary Fig 1. Study criteria in derivation cohort

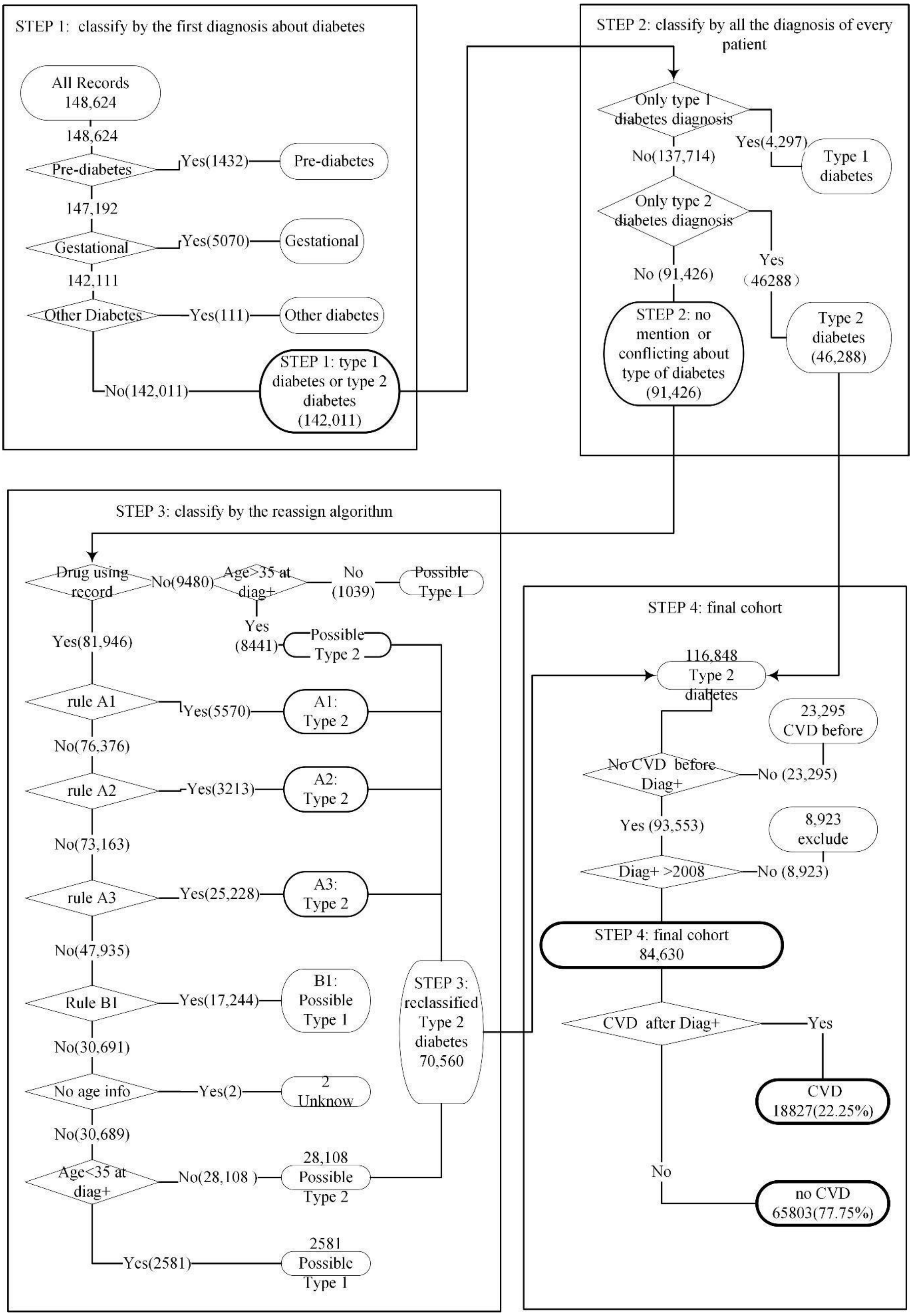

Rule A1: Reassign anyone as type 2 if they have prolonged use of Sulfonylureas for more than 12 months use over time. Rule A2. Reassign anyone as type 2 of they are clearly observable for drugs for long periods after diagnosis, but

are clearly not on insulin. Rule A3. Reassign anyone as type 2 of patients who are clearly observable for drugs in the 1year period after diagnosis, but do not have insulin prescribed in that period. Rule B1. Reassign anyone as type 1 who are clearly observable for drugs in the 1year period after diagnosis, and have insulin prescribed in that period.
